# How High-Flow Nasal Cannula Is Impacted by Nasogastric/Esophageal Tube: A Bench Study

**DOI:** 10.1097/CCE.0000000000001325

**Published:** 2025-09-30

**Authors:** Fernando Vieira, Annia Schreiber, Mayson L. A. Sousa, Rosie Butterworth, Shreyas Bhor, Antenor Rodrigues, Vorakamol Phoophiboon, Matthew Ko, Laurent Brochard

**Affiliations:** 1 Critical Care, Keenan Centre for Biomedical Research, Li Ka Shing Knowledge Institute, St. Michael’s Hospital, Toronto, ON, Canada.; 2 Interdepartmental Division of Critical Care Medicine, University of Toronto, Toronto, ON, Canada.; 3 Fisher & Paykel Healthcare Ltd., Auckland, New Zealand.; 4 Division of Critical Care Medicine, Department of Medicine, Faculty of Medicine, Chulalongkorn University, Bangkok, Thailand.; 5 Department of Respiratory Therapy, College of Rehabilitation Sciences, Rady Faculty of Health Sciences, University of Manitoba, Winnipeg, MB, Canada.

**Keywords:** gastric feeding tube, indwelling, nasal cannula, respiratory therapy

## Abstract

High-flow nasal cannula (HFNC) is a common noninvasive respiratory therapy for respiratory failure, offering positive airway pressure and dead space clearance. In critically ill patients, additional nasal tubes for feeding or monitoring are often required, but their effect on HFNC performance is not well understood. This bench study evaluated the impact of nasal tube placement on dead space clearance, nasopharyngeal pressure, and airway resistance using standard and asymmetrical cannulas. Inserting a 5-Fr tube with a standard cannula had very little effect, whereas bigger sizes (12-Fr and 16-Fr) slightly increased airway pressure and reduce Co_2_ clearance. An asymmetrical cannula exhibited variable effects depending on the side of the tube placement. Higher pressure and better clearance were achieved with a tube placed in the smaller cannula side. However, if occlusion of the two nares becomes excessive with the tube in place, downsizing the cannula might be recommended.

## To the Editor:

High-flow nasal cannula (HFNC) is a noninvasive respiratory support technique that is now widely indicated for patients with respiratory failure (1, 2). Its primary mechanisms of action stem from the delivery of heated and humidified high-flow gas and include the clearance of anatomical dead space and the generation of positive airway pressure (3). We previously demonstrated that HFNC also increases expiratory resistance, which may contribute to the observed reduction in respiratory rate (4). Overall, HFNC has been shown to improve oxygenation, reduce respiratory rate, and decrease inspiratory effort (5, 6).

Acutely ill patients often require additional tubes within the nasal cavity for various clinical purposes, including nasogastric feeding or suction tubes, as well as esophageal catheters for respiratory monitoring. Previous studies assessed the physiologic effects of HFNC using esophageal manometry (4, 7). However, it remains unclear whether, and to what extent, the presence of a tube introduced through the nose might influence the effects of HFNC. The concomitant use of HFNC and these additional tube could potentially impact HFNC efficacy.

We performed a bench study to evaluate the effect of placing an additional tube(nasogastric or esophageal) through the nares on the physiologic effects of HFNC using a previously published model (4). Specifically, we assessed the effects on rebreathing volume (dead space clearance capacity), end-expiratory nasopharyngeal pressure (NasoP), and inspiratory and expiratory resistance using two models of nasal cannula (standard and asymmetrical).

For the current bench study, we used two different manikin heads with upper airways: 1) Laerdal Medical (Stavanger, Norway)—a standardized, commercially available airway simulator made from silicone for medical training (softer and less anatomically detailed; total cross-sectional area of the nares = 157 mm^2^) and 2) a human 3D-printed airway manikin (Fisher & Paykel Healthcare, Auckland, New Zealand)—created from medical imaging using resins, providing a realistic representation of airway anatomy, primarily used for research but more rigid than real conditions (total cross-sectional area of nares = 98 mm^2^). Each manikin head was connected to an active breathing simulator (ASL-5000; IngMar Medical, Pittsburgh, PA) operating in flow-controlled pump mode, set to generate a tidal volume of 450 mL at a respiratory rate of 30 beats/min, with an inspiratory-to-expiratory ratio of 1:2. The manikin mouths were kept closed, and a constant Co_2_ production of 300 mL/min arriving in the upper lung was simulated by delivering 5% Co_2_ at 6 L/min into the breathing simulator. We tested three tube sizes: 5-Fr, 12-Fr, and 16-Fr. The 5-Fr tube was the only size tested in both airways, since the rigid 3D-printed airway did not allow the 12-Fr and 16-Fr tubes. The HFNC flow rate was set at 0, 20, 40, and 60 L/min (2 min each). We used two nasal interfaces for HFNC: standard, symmetrical (OPT944-Optiflow+; Fisher & Paykel Healthcare), and asymmetrical (OPT964-Optiflow+ Duet; Fisher & Paykel Healthcare), both in size M. For the asymmetrical cannula, the tube was inserted on both the larger and smaller cannula sides. We measured end-expiratory NasoP, inspiratory and expiratory airway resistance, and capnography at the trachea level, recorded at 256 Hz using the FluxMed System (FluxMed GrE, MBMED, Buenos Aires, Argentina). To examine the effect of an additional tube during HFNC therapy—considering tube size and flow rate variations—we performed a two-way analysis of variance with a post hoc Bonferroni test, considering statistical significance at *p* value of less than 0.05.

**Figure 1.**
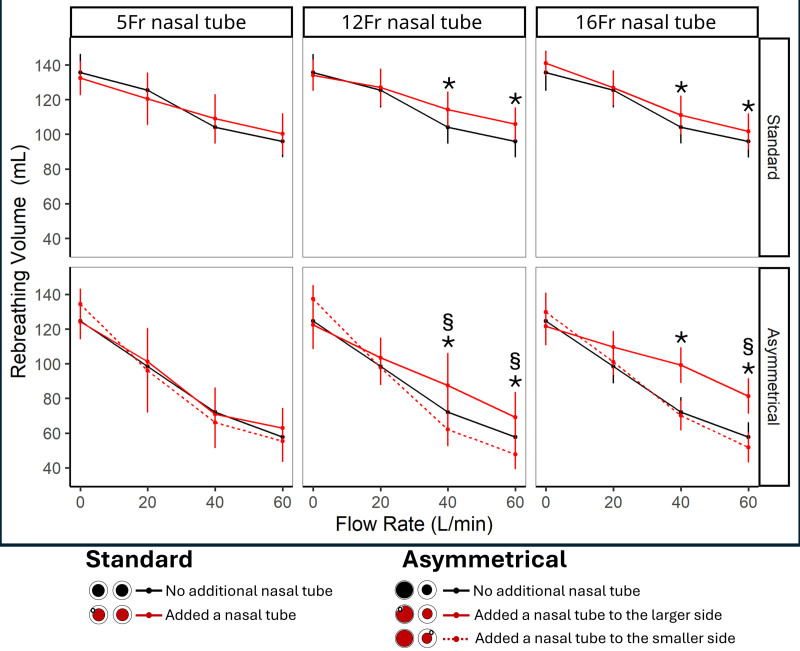
Impact of an additional nasal tube placement in the nasal cavity on rebreathing volume during high-flow nasal cannula therapy. Two-way analysis of variance and post hoc Bonferroni test (*p* < 0.05) were used for comparisons. *Additional nasal tube (standard or large side asymmetrical) vs. none. §Additional nasal tube (smaller side asymmetrical) vs. none.

**Figure 2.**
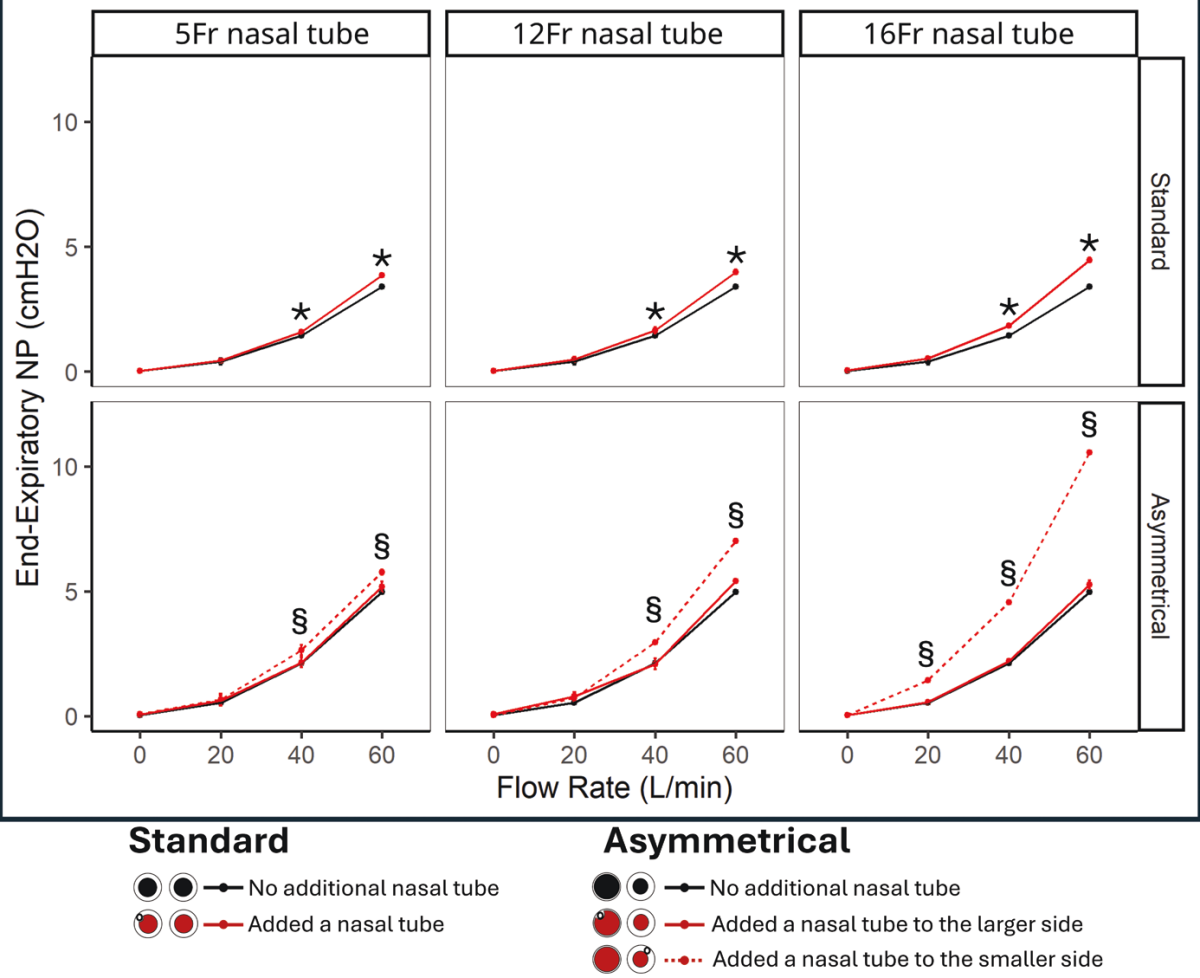
Impact of an additional nasal tube placement in the nasal cavity on end-expiratory nasopharyngeal pressure (NP) during high-flow nasal cannula therapy. Two-way analysis of variance and post hoc Bonferroni test (*p* < 0.05) were used for comparisons. *Additional nasal tube (standard and large side asymmetrical) vs. none. §Additional nasal tube (smaller side asymmetrical) vs. none.

**Figure 3.**
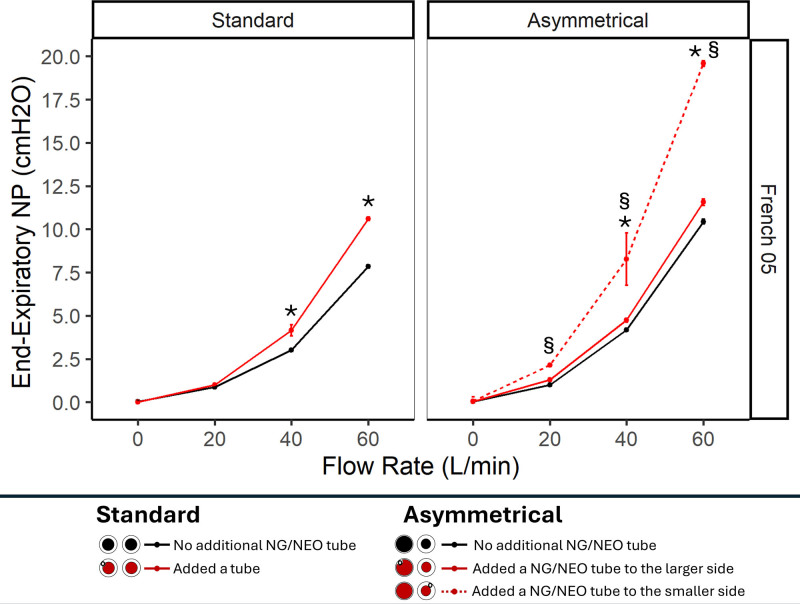
Five-Fr nasal tube on the 3D-printed airway: impact on end-expiratory nasopharyngeal pressure (NP) during high-flow nasal cannula therapy. Two-way analysis of variance and post hoc Bonferroni test (*p* < 0.05) were used for comparisons. *Additional nasal tube (standard and large side asymmetrical) vs. none. §Additional nasal tube (smaller side asymmetrical) vs. none.

Using the standard cannula, dead space clearance did not change with the insertion of a 5-Fr tube but was slightly reduced with bigger sizes, represented by an increase in rebreathing volume (**Fig. 1**). It also led to a small increase in end-expiratory NasoP (**Figs. 2** and **3**), inspiratory resistance (5-Fr in 3D-printed airway: 11.2 ± 0.06 vs. 8.2 ± 0.05 at 60 L/min; *p* < 0.001) and expiratory resistance (5-Fr in 3D-printed airway: 23.2 ± 0.16 vs. 15.5 ± 0.11 at 60 L/min; *p* < 0.001), compared with the absence of a tube.

Using the asymmetrical cannula, effects were different according to the side of the tube inserted into the nasal cavity. Inserting the tube on the side of the smaller cannula tip resulted in the highest end-expiratory NasoP (Figs. 2 and 3), inspiratory (5-Fr in 3D-printed airway: 18.9 ± 0.15 vs. 11.73 ± 0.1 at 60 L/min; *p* < 0.001) and expiratory (5-Fr in 3D-printed airway: 52.5 ± 0.47 vs. 31.8 ± 0.28 at 60 L/min; *p* ≤ 0.001) breathing resistances but it also decreased the rebreathing volume, suggesting a more efficient Co_2_clearance of the upper airway (Fig. 1). In contrast, placing the tube on the nose side with larger nasal cannula size resulted in no significant impact on end-expiratory NasoP and breathing resistances, while significantly reducing the HFNCs dead space clearance effect and increasing rebreathing volume.

This bench study suggests that the concurrent use of a nasal tube with HFNC has a minimal impact with a 5-Fr tube in general but can significantly alter its physiologic effects with larger tubes. Indwelling a tube with a standard cannula reduced upper airway Co_2_ clearance (increase in rebreathing volume) and raised NasoP, as well as both inspiratory and expiratory resistances. Inserting the tube with an asymmetrical cannula produced different effects depending on the side of insertion: on the side with the larger cannula tip, the primary effect was reduced Co_2_ clearance, while on the side with the smaller cannula, an increase in end-expiratory NasoP, breathing resistance, and Co_2_ clearance. Higher occlusion in the nasal cavity, caused by a thicker tube and/or smaller nares, led to more pronounced modifications in the physiologic effects of HFNC. This was most evidenced when the smallest tested tube (5-Fr) was inserted into the 3D-printed airway (smaller and rigid airway) (Fig. 3). However, the 5-Fr on the non-3D-printed airway has a nonclinically relevant effect despite some statistically significant differences. While recent trials have demonstrated HFNCs noninferiority to noninvasive ventilation for various types of respiratory failure (8, 9), the clinical efficacy of HFNC can be significantly impacted by concurrent nasal interventions. In critical care settings, it is common for patients receiving HFNC to have an additional nasal tube inserted, such as feeding tubes. Our results are showing that a concurrent use of nasal tube can modify the physiologic effects of HFNC by affecting the delivery of positive pressure, impacting upper airway clearance, and altering airway resistance. Understanding how these factors interact is essential for optimizing HFNC therapy and ensuring its benefits are not compromised by unintended physiologic changes.

It is already known that the airflow circulation is responsible for the dead space clearance, and it is dependent upon the nares’ orifices left unobstructed. Furthermore, asymmetrical nasal cannulas alter the airflow dynamics and enhance Co_2_ clearance by creating an asymmetrical occlusion, directing airflow into one nostril and out through the opposite side, purging out Co_2_ (10). Therefore, adding a nasal tube may further modify the airflow during expiration, impacting both the upstream pressure in the nasopharynx and the efficacy of dead space clearance. Since the occlusion promoted by a nasal tube does not deliver flow but instead partially folds the adjacent side of the nasal cannula, it redirects airflow toward the contralateral nostril, further affecting dead space clearance.

Considering dead space clearance and positive airway pressure as the primary effects of HFNC, positioning the tube on the smaller side of an asymmetrical cannula generates higher pressure and improves Co_2_ clearance efficiency. However, caution is needed with this approach, as excessive occlusion may occur. Therefore, when the additional tube is indwelling with the smaller cannula tip side, we recommend downsizing the cannula or using a symmetrical cannula. Alternatively, when using a standard cannula, adding a concurrent tube can increase pressure, while posing a risk of reducing the dead space clearance efficiency. These findings require validation in clinical settings to ensure their accuracy and practical applicability.
